# Supplementary material for: Comparative multiomics analysis of cell physiological state after culture in a basket bioreactor
Source: Sci Rep. 2022 Nov 23;12:20161. doi: 10.1038/s41598-022-24687-4 (PMC9686226; doi:10.1038/s41598-022-24687-4)
Supplement: Supplementary file 1 — Supplementary Information 1. [file 41598_2022_24687_MOESM1_ESM.zip › raw data/Transcriptomic raw data/6.Enrichment/1.GO/TE1vsTC1/GSEA/TC1/DNA_POLYMERASE_ACTIVITY/DNA_POLYMERASE_ACTIVITY(GO_0034061).html]

Details for gene set DNA\_POLYMERASE\_ACTIVITY(GO:0034061)[GSEA]


|  || Dataset | readcount.sample |
| Phenotype | sample.cls |
| Upregulated in class | TC1 |
| GeneSet | DNA\_POLYMERASE\_ACTIVITY(GO:0034061) |
| Enrichment Score (ES) | -0.28060928 |
| Normalized Enrichment Score (NES) | -1.0 |
| Nominal p-value | 0.0 |
| FDR q-value | 1.0 |
| FWER p-Value | 1.0 |
Table: GSEA Results Summary

  

Fig 1: Enrichment plot: DNA\_POLYMERASE\_ACTIVITY(GO:0034061)
  
Profile of the Running ES Score & Positions of GeneSet Members on the Rank Ordered List

  

| PROBE | DESCRIPTION   (from dataset) | GENE SYMBOL | GENE\_TITLE | RANK IN GENE LIST | RANK METRIC SCORE | RUNNING ES | CORE ENRICHMENT || 1 | 103242185 | MYBBP1A |  |  | 6282 | 1.275 | -0.1870 | Yes |
| 2 | 103216324 | DNTT |  |  | 6716 | 1.242 | -0.1151 | Yes |
| 3 | 103228941 | POLE2 |  |  | 7684 | 1.167 | -0.0726 | Yes |
| 4 | 103231730 | POLA1 |  |  | 8900 | 1.092 | -0.0467 | Yes |
| 5 | 103231149 | POLG |  |  | 9373 | 1.061 | 0.0102 | Yes |
| 6 | 103215689 | POLB |  |  | 10637 | 0.982 | 0.0258 | Yes |
| 7 | 103239455 | POLE |  |  | 11657 | 0.916 | 0.0476 | Yes |
| 8 | 103226140 | POLD2 |  |  | 12844 | 0.842 | 0.0564 | Yes |
| 9 | 103228671 | POLQ |  |  | 13089 | 0.828 | 0.1063 | Yes |
| 10 | 103229871 | POLA2 |  |  | 13330 | 0.812 | 0.1552 | Yes |
| 11 | 103216413 | POLL |  |  | 14509 | 0.739 | 0.1568 | Yes |
| 12 | 103226146 | POLM |  |  | 14598 | 0.733 | 0.2067 | Yes |
| 13 | 103246469 | POLN |  |  | 15682 | 0.663 | 0.2070 | Yes |
| 14 | 119627160 | LOC119627160 |  |  | 17259 | 0.553 | 0.1773 | No |
| 15 | 103214928 | TERT |  |  | 17857 | 0.503 | 0.1875 | No |
| 16 | 119623175 | LOC119623175 |  |  | 20403 | 0.210 | 0.0893 | No |
Table: GSEA details
[plain text format]

  

Fig 2: DNA\_POLYMERASE\_ACTIVITY(GO:0034061)
  
Blue-Pink O' Gram in the Space of the Analyzed GeneSet

  

Fig 3: DNA\_POLYMERASE\_ACTIVITY(GO:0034061): Random ES distribution
  
Gene set null distribution of ES for
 **DNA\_POLYMERASE\_ACTIVITY(GO:0034061)**
